# Medical Society of the County of Erie

**Published:** 1900-02

**Authors:** F. C. Gram


### Society Proceedings.

### MEDICAL SOCIETY OF THE COUNTY OF ERIE.

*Seventy-ninth Annual Meeting, held January 9, 1900.*

REPORTED BY F. C. GRAM, M. D., Secretary.

THE Medical Society of the County of Erie held its seventy-ninth annual meeting in the parlors of the Young Men's Christian Association, Mohawk and Pearl Streets, Buffalo, Tuesday, January 9, 1900.

The president, Dr. J. B. COAKLEY called the assembly to order at 9.30 a.m., whereupon the minutes of the semi-annual meeting, and of the memorial meeting, held on the death of Dr. W. C. Earl, were read and approved.

Dr. W. W. POTTER, chairman of the committee on membership, presented the names of the following applicants and recommended their admission into the society: Drs. Felix Hintz, Catherine E. Kelly, Daniel P. Doyle, William J. Deane, James W. Nash and Ernest W. Ewell. The report was received and on motion the applicants were elected to membership.

Applications for membership were received from the following: Drs. Chauncey P. Smith and William R. Little.

Dr. W. W. POTTER offered amendments to the by-laws relative to the admission of members by striking out the words "who shall present it at a regular meeting to the society," after the words "shall make his application to the secretary," in Section 1, Article VII., making the revised section read:

Section 1. Every physician or surgeon residing in the County of Erie, who may hereafter wish to become a member of this society, shall make his application to the secretary. It shall be referred to the committee on membership, which shall report the result of its investigation, accompanied by a recommendation for or against the applicant, at the next regular meeting of the society. The applicant shall be admitted upon a majority vote of the members present.

Also by striking out the words "but shall not be acted upon until the next regular meeting," after the words "shall be presented at a regular meeting," in Sec. 5, Article VII., making it read:

Section 5. Applications for admission to the society shall be accompanied by the initiation fee of $2.00, and shall be presented at a regular meeting. The application shall be signed by the applicant and shall be made in the following form.

Dr. Potter explained that the by-laws were originally framed as at present to afford ample time for investigation of the applicant's qualifications, but under the new state law this is rendered unnecessary, as he is able to establish his identity and exhibit his credentials at once, or not at all. The proposed amendments were laid over under the rules until the next regular meeting.

The president appointed Drs. Wm. C. Krauss, E. Wende and F. E. Fronczak a committee to nominate officers for the ensuing year :

The committee presented the following nominations : For president, Dr. E. H. Ballou, of Gardenville; for vice-president, Dr. Wm. C. Phelps; for secretary, Dr. Franklin C. Gram; for treasurer, Dr. Edward Clark ; for librarian, Dr. W. C. Callanan; for board of censors, Drs. J. B. Coakley, chairman, H. R. Hopkins, I. W. Potter, T. F. Dwyer and F. E. Fronczak; for committee on membership, Drs. W. W. Potter, chairman, G. W. McPherson and J. J. Walsh.

On motion the secretary was directed to cast the ballot of the society for the nominees. This being done they were declared unanimously elected.

All the delegates to the state society were requested to attend the annual meeting at Albany, January 30, 31, and February 1, 1900. and qualify for permanent membership.

On motion of Dr. J. H. Grant the secretary was authorised to publish a new edition of the by-laws of the society, with a revised list of membership, and mail a copy to each member.

The following report from the Board of Censors was read :

To the Medical Society of the County of Erie :

Your board of censors respectfully reports that since its last report made to the society at its semi-annual meeting held on June 13, 1899, it has, with the aid of our counsel, Hon. Tracy C. Becker, investigated and prosecuted the following cases of persons unlawfully practising medicine or surgery in this county.

In the case of Stephen T. Lee, who, at the date of our last report, had been arrested and brought before the Police Court on the charge of administering for hire treatment and medicine to many persons afflicted with cancer and other diseases, the said Lee not being licensed to practise medicine and surgery, he was duly held to answer before the Grand Jury, and was thereafter indicted; and on the 29th day of June, 1899, he was convicted in the County Court of Erie County and fined $100; which sum, upon order of the court, and pursuant to the statute, was paid by the county treasurer to the treasurer of this society. As Lee promised to abstain from further illegal practice, this board has refrained from instituting any civil suits against him for penalties; but it has him under constant surveillance and will institute proceedings against him in case he shall be guilty of any further infraction of the law.

Ferdinand Szymanski was indicted upon the complaint of this board for practising medicine without a license, and on December 7, 1899, was convicted by the verdict of a jury at the criminal trial term of the Supreme Court. The court believing his statement that he was ignorant of the provisions of our laws, and that he thought he could lawfully prescribe for cases if he did so without charging therefor a fee, suspended sentence on him. This man is still under the surveillance of your board and its agents and will be further prosecuted or brought up for sentence on his conviction if he should again attempt to practice medicine.

In the case of the so-called christian science healer, George H. Kinter, and James C. Saunders, mentioned in our last report, these persons were held to answer at the September term of the United States District Court for the crime of manslaughter, in having negligently caused the death of Rolf Saunders, the minor child of the said James C. Saunders, at the United States Fort Porter, in this city. The counsel for this society assisted and advised with Assistant United States District Attorneys Brown and Dudley concerning the case. Practically the same evidence as was given before the United States Commissioner was presented to the Grand Jury. Diligent effort has been made to procure other evidence which should tend to show clearly that the Saunders child exhibited symptoms and appearances of the disease of which he died, viz.: double pneumonia, so manifestly as to make it criminal negligence for the child's father and healer Kinter to fail to call in a licensed physician who could properly diagnose and treat the case. This evidence could not be procured, chiefly because all the persons who had an opportunity to observe the child during its illness, to-wit: the father and mother of the child and healer Kinter, and his wife having been arrested by order of former United States District Attorney Close and charged with this crime, could not be compelled to give testimony in the case. Moreover, Acting District Attorney Brown and United States District Judge Coxe entertained doubts as to whether the provisions of the United States statutes defining the crime of manslaughter where broad enough to embrace a case of death due to mere acts of negligent omission of duty such as were alleged to exist in this case. After careful consideration of these difficulties and full consultation between the United States authorities, our counsel and Judge Coxe, it was reluctantly deemed best that the United States Grand Jury should be advised not to find a bill of indictment against Saunders and Kinter, because it was believed that if a bill had been found no conviction could have been obtained.

In the matter of the amendment to the ordinances of the City of Buffalo, proposed by Health Commissioner Wende, so to require in definite terms, that all other persons, as well as physicians, in charge or in attendance upon cases of contagious or infectious disease should report the same to the Board of Health, your Board, its counsel and many members of the society attended upon and presented arguments before the Board of Aldermen and the Board of Councilmen and their committees on many occasions. The amendment to the ordinance as originally drafted included by specific mention christian science healers as well as midwives and nurses. The whole body of christian scientists in this city at first violently opposed, personally and by able counsel, any amendments to the ordinance whatever, but later, having been driven from this position, they shifted their opposition to the claim that they should not be specifically mentioned and should not be put in the same category with nurses and midwives. This amendment, however, changed so as to omit the specific mention of any particular class of persons, but so as to include everyone who might be in charge of or in attendance upon persons afflicted with contagious or infectious diseases, was finally adopted by both branches of the Common Council, was approved by his honor, the Mayor, and became a local law.

This Board has also investigated a number of cases of violation of medical ethics by physicians who advertise, etc., and has remonstrated with them; and in several cases our remonstrances have been heeded and such practises discontinued. It has also warned one graduate in medicine that he had neglected to register in the County Clerk's office as a physician, and he has complied with the law in this respect. It has now under investigation and consideration a very flagrant and long standing case of practising medicine under an assumed name and of advertising in the newspapers and sending circulars through the mails for fraudulent purposes. Our counsel is in communication with the State Board of Medical Examiners and the State Board of Regents and has consulted with other public authorities in reference to this case. He expects soon to be able to institute a vigorous prosecution of the offender, not only to have his license as a physician revoked but also to have him indicted and punished for using the mails for fraudulent purposes, an offense of which the person in question has once before been con victed and fined in the United States Court.

In conclusion this board respectfully recommends that the same compensation per annum now allowed our counsel, Hon. Tracy C. Becker, be paid to him on the order of the board. We also recommend that this board be given authority to employ such detectives and other agents as may be required for the performance of its duties, and to draw upon the treasurer, as heretofore for their reasonable compensation.

Respectfully submitted,
J. B. COAKLEY, Chairman.
FRANCIS E. FRONCZAK.
IRVING W. POTTER.

Buffalo, N. Y., January 9, 1900.

On motion of Dr. CALLANAN the report was received and the recommendations therein adopted.

The treasurer, Dr. EDWARD CLARK, submitted his annual report as follows:

| | | |
|---|---|---|
| Cash on hand, January 1, 1899 | $159.00 | |
| Collected during year | 347.00 | |
| Fine of S. T. Lee | 100.00 | |
| | | $606.00 |
| Disbursements as per itemised account | $460.37 | |
| Balance on hand, Dec. 31, 1899 | 145.63 | |
| | | $606.00 |

Referred to Drs. Irving W. Potter and Wyckoff for audit, who later reported that they had found the vouchers and accounts correct.

Treasurer CLARK reported that four of the suspended members, Drs. Bartow, Wheeler, Watkins and Baker, had paid up their arrears, and on motion of Drs. Callanan and Potter they were reinstated. A question concerning Dr. Baker was referred to the Board of Censors.

The librarian, Dr. CALLANAN, submitted a very gratifying annual report. He stated that for the past five years the books have been located in the University of Buffalo and are cared for by the librarian of the University. His request to members for donations of books, especially such as will be valuable for future reference, met with a number of responses, the most generous and valuable coming from Dr. Thomas M. Johnson, who donated 112 volumes, among which were a number giving some of the earlier and later history of medicine in this state through transactions of medical societies.

A vote of thanks was tendered Dr. Johnson for his valuable gift.

Dr. C. E. CONGDON called attention to the antivivisection movement.

Dr. W. W. POTTER stated that the president of the American Medical Association had addressed a circular letter on this subject to all medical bodies and moved that this question be referred with power to the legislative committee of this society. The motion was adopted.

Dr. H. R. HOPKINS offered the following resolution:

*Resolved*, That the Medical Society of the County of Erie approves of the project of state care in state sanitoria of the poor and others suffering with early and therefore curable consumption; and that we bespeak for this project the confidence and active support of the medical society of the State of New York, and of our representatives, legislative and executive, at Albany, to the end that suitable legislation at the earliest practicable moment may test our beliefs and expectations for the betterment of an afflicted, neglected and dangerous class of our people.

*Resolved*, That this resolution, duly attested by our president and secretary, and the seal of this society, be transmitted to the society and persons above mentioned.

The resolutions were adopted and referred to the legislative committee with power to further the spirit of the same at Albany whenever the subject shall come up for action before the legislature.

Dr. BENTLEY S. BOURNE, of Hamburg, offered the following memorial, which was adopted:

WHEREAS, In the death of Dr. W. E. Robbins the Medical Society of the County of Erie is again called upon to record the name of a member deceased, taken in the prime of a life giving bright promise of long years of coming usefulness. He was stricken suddenly, and while in active duty the summons came.

God's finger touched him and he slept.

*Resolved*, That we bow in reverent submission to the divine mandate of Him who doeth all things well.

*Resolved*, That in his death this society has lost a valuable member, that we deplore his untimely removal from us and will ever cherish his memory with respect.

*Resolved*, That the sympathy of this society be extended to his widow and daughters, with a copy of these resolutions.

*Resolved*, That these resolutions be entered on the minutes of this society and given to the BUFFALO MEDICAL JOURNAL for publication.

The vice-president, Dr. BALLOU, was called to the chair, whereupon, Dr. Coakley read the president's annual address on Water supply and sewerage system of Buffalo, which will be published in the JOURNAL for March.

Dr. COAKLEY stated that invitations had been sent to our city officials, as well as to the health and executive officers of Tonawanda, Niagara Falls and Lockport, and invited those present to participate in the discussion.

Dr. BENEDICT stated that our water supply was purer than that of most cities, and he would not charge all typhoid to the water, but much to local infection. He believed that Buffalo ought to extend its mains and sell water to adjacent towns.

Commissioner of Public Works, Adam Boeckel, said he became a member of the board of aldermen in 1892, and always took an active interest in all that pertained to public health.

The phenomenal growth of American cities necessitated constant improvement of many things which were begun in a very primitive way. He believed in the scientific disposal of sewage and hoped that the Medical Society of the County of Erie and similar societies, would continue to agitate this question until not only local or state, but national laws are enacted compelling every city to take care of its sewage, without polluting streams or bodies of water and endangering the lives of neighboring towns. He declared himself ready at all times officially and personally to aid in any well directed movement toward improving our system of water supply and especially to prevent its pollution.

Commissioner of Public Works, M. J. Healy, chairman of the board, considered a general discussion of a subject like this of the greatest value. Scientific bodies should be active and aggressive. It may not be practical politics, but when the public understands that it means the saving of human lives, it will give a hearty approval. While our water system is one of the best in the country, the board of public works has seriously considered many improvements which become more important as we investigate. He pointed out what improvements were intended in the water supply and sewerage systems, and said the board recognises the rights and duties of the medical profession in this matter, and would at all times be glad to consult with and receive the advice and coöperation of its members.

Chief Engineer Knapp, of the water department, explained on a map the source of the city's present water supply and how it would be possible to obtain a still purer supply at a moderate cost.

City Engineer Bardol explained the sewer system of Buffalo, on a map.

Dr. W. A. Scott, health officer of Niagara Falls, said the subject was of vital interest to his city, as the Buffalo sewage finds its way to its doors. All sewage should be so disposed of as not to contaminate the water supply of municipalities. He presented a list of 1,117 cases of infectious diseases occurring in Niagara Falls during the past five years, of which 906 were typhoid fever, and probably one-third actual cases of typhoid had not even been reported. The source of this disease was unquestionably a polluted water supply, caused by the sewage of Buffalo.

Health Commissioner Ernest Wende, of Buffalo, stated that since no more water had been taken through the Bird Island inlet the typhoid fever rate had been reduced one-half. He briefly explained how this was brought about and said that the bacteriological department, which had been so unmercifully criticised and even ridiculed, was the means of saving hundreds of lives annually.

Drs. S. S. Greene, H. R. Hopkins, W. W. Potter, S. W. Wetmore and city bacteriologist Bissell briefly discussed the subject.

Papers were read on The diagnosis of tumors of the breast; its importance, by Dr. Chauncey P. Smith; Neuroses of childhood, by Dr. Maud J. Frye; Unusual condition of the skin and kidneys, following an operation for appendicitis, by Dr. W. G. Taylor. These papers will be printed in a future issue of the JOURNAL.

A vote of thanks was tendered the retiring officers and all the essayists; also to the Young Men's Christian Association for the free use of its parlors.

On motion the usual honorarium of $50 each was voted to the secretary and treasurer for services during the past year.

The chair appointed the following committee on legislation: Drs. Ernest Wende, Edward Clark and W. W. Potter.

The president elect, Dr. E. H. Ballou, assumed the chair and at 1.10 p. m. the society adjourned..

